# Extracellular vesicle release and uptake by the liver under normo- and hyperlipidemia

**DOI:** 10.1007/s00018-021-03969-6

**Published:** 2021-10-19

**Authors:** Krisztina Németh, Zoltán Varga, Dorina Lenzinger, Tamás Visnovitz, Anna Koncz, Nikolett Hegedűs, Ágnes Kittel, Domokos Máthé, Krisztián Szigeti, Péter Lőrincz, Clodagh O’Neill, Róisín Dwyer, Zhonglin Liu, Edit I. Buzás, Viola Tamási

**Affiliations:** 1 Department of Genetics, Cell- and Immunobiology, Semmelweis University, Budapest, Hungary; 2 Research Centre for Natural Sciences, Institute of Materials and Environmental Chemistry, Budapest, Hungary; 3 Department of Biophysics and Radiation Biology, Semmelweis University, Budapest, Hungary; 4 Eötvös Loránd Research Network, Institute of Experimental Medicine, Budapest, Hungary; 5 Hungarian Centre of Excellence for Molecular Medicine, In Vivo Imaging ACF, Budapest, Hungary; 6 Department of Anatomy, Cell and Developmental Biology, Eötvös Loránd University, Budapest, Hungary; 7 Discipline of Surgery, National University of Ireland, Lambe Institute for Translational Research, Galway, Ireland; 8 Department of Medical Imaging, University of Arizona, Tucson, AZ USA; 9 Hungarian Centre of Excellence for Molecular Medicine, Semmelweis University Extracellular Vesicle Research Group, Budapest, Hungary; 10 ELKH-SE Immune-Proteogenomics Research Group, Budapest, Hungary

**Keywords:** Extracellular vesicle, Extracellular particles, Hepatocyte, Kupffer cell, Liver sinusoidal endothelial cells

## Abstract

Liver plays a central role in elimination of circulating extracellular vesicles (EVs), and it also significantly contributes to EV release. However, the involvement of the different liver cell populations remains unknown. Here, we investigated EV uptake and release both in normolipemia and hyperlipidemia. C57BL/6 mice were kept on high fat diet for 20–30 weeks before circulating EV profiles were determined. In addition, control mice were intravenously injected with ^99m^Tc-HYNIC-Duramycin labeled EVs, and an hour later, biodistribution was analyzed by SPECT/CT. In vitro*,* isolated liver cell types were tested for EV release and uptake with/without prior fatty acid treatment. We detected an elevated circulating EV number after the high fat diet. To clarify the differential involvement of liver cell types, we carried out in vitro experiments. We found an increased release of EVs by primary hepatocytes at concentrations of fatty acids comparable to what is characteristic for hyperlipidemia. When investigating EV biodistribution with ^99m^Tc-labeled EVs, we detected EV accumulation primarily in the liver upon intravenous injection of mice with medium (326.3 ± 19.8 nm) and small EVs (130.5 ± 5.8 nm). In vitro*,* we found that medium and small EVs were preferentially taken up by Kupffer cells, and liver sinusoidal endothelial cells, respectively. Finally, we demonstrated that in hyperlipidemia, there was a decreased EV uptake both by Kupffer cells and liver sinusoidal endothelial cells. Our data suggest that hyperlipidema increases the release and reduces the uptake of EVs by liver cells. We also provide evidence for a size-dependent differential EV uptake by the different cell types of the liver. The EV radiolabeling protocol using ^99m^Tc-Duramycin may provide a fast and simple labeling approach for SPECT/CT imaging of EVs biodistribution.

**Supplementary Information:**

The online version contains supplementary material available at 10.1007/s00018-021-03969-6.

## Introduction

Extracellular vesicles (EV) are mostly spherical, phospholipid bilayer-enclosed particles [1]. They were originally thought to be carriers for the removal of cellular waste [2]. However, we now know that due to their complex molecular composition (including nucleic acids, lipids, proteins and carbohydrates), they are significant players of cell–cell communication [3, 4]. The composition of EVs is determined by the types and functional states of the donor cells. The versatile roles of EVs have been confirmed in several physiological and pathological processes [5]. Even though the classification of EVs is based on their biogenesis, often it is not possible to determine the biogenetic origin of EVs. Size-based subfractions of EVs include large EVs (lEVs, > 1 µm), medium EVs (mEVs, 150 nm to 1 µm) and small EVs (sEVs ≤ 100 nm) [6].

Biodistribution studies of EVs have shown that they primarily accumulate in liver and spleen [7–13]. According to Imai and co-workers, even after depletion of hepatic macrophages (Kupffer cells, KCs), EVs accumulated in the liver [12]. This suggests that liver cell populations other than KCs are also involved in EV removal from the circulation. Both parenchymal hepatocytes (HEPs) and non-parenchymal cells (NPCs) are capable of endocytosis suggesting a potential strong EV scavenger function liver cells in the body [14]. Although the rapid clearance of EVs by liver has already been demonstrated [7–12], the relative contribution of the individual liver cell types to EV release and elimination has not been investigated yet.

Currently, over 3 million adults (2020) have been diagnosed with primary or secondary hyperlipidemia in Europe and in the United States, with the number of cases increasing dramatically [15]. Cardiovascular diseases often associated with hyperlipidemia represent the number one cause of death worldwide [16]. The high-fat Western diet is known to increase the likelihood of developing secondary, diet-induced hyperlipidemia. We were compelled to carry out this study, because we hypothesized that the level of circulating lipoprotein particles may have an impact on EV release and uptake by the liver. Of note, higher blood lipid levels may lead to lipotoxicity, which, in turn, may also alter the dynamics of EV production by liver cells [17–19].

Here, we report that hyperlipidemia indeed has a major impact on the dynamics of hepatic EV production and uptake. These results may have significant implications for the development of EV-based therapies.

## Materials and methods

### Animal experiments

All mouse experiments followed the European Union’s Council Directive (86/609/EEC), and were approved by the Semmelweis University’s Institutional Animal Care and Use Committee (PE/EA/1655-7/2018). Male, C57BL/6 mice were housed in a temperature-controlled (25 °C) room, with normal light cycle (12–12 h), and were given free access to food and water. At the age of 6 weeks, mice were divided into two groups and were fed either by a high fat diet (HFD, 45 kcal% fat, D12451, Research Diets, USA) or by a normal mouse diet for up to 20 and 30 weeks (HFD-20 and HFD-30, respectively). Throughout the experimental period, the body weight was measured two-weekly. At the end of the high fat/normal diet, mice were fasted for five hours prior to glucose tolerance test. The glucose levels were measured using a Dcont Ideal instrument (Dcont, Hungary) from blood obtained by cutting off the end of the tail before and 30 and 90 min after the intraperitoneal injections of glucose (20 w/v%, 2 g/kg). Plasma levels of low-density lipoprotein cholesterol (LDL-C)/ very low-density lipoprotein cholesterol (VLDL-C) and high-density lipoprotein cholesterol (HDL-C) were determined with a HDL and LDL/VLDL Quantification Kit (Sigma, USA). Hepatic triglyceride levels were measured using a Triglyceride Colometric Assay Kit (Cayman Chemical, USA). For total RNA, 40–50 mg pieces of the liver were homogenized with a Micropestle (Geneaid, Taiwan) in QIAzol Lysis Reagent (QIAGEN, Germany), and total RNA was isolated with a Blood/Cell Total RNA Mini Kit (Geneaid, Taiwan). cDNA was synthesized using SensiFAST cDNA Synthesis Kit (Bioline, UK), from 1 µg of total RNA. Real-time quantitative PCR (RT-qPCR) was performed using a 7900 HT Fast Real-Time PCR System (Applied Biosystems, USA), using SensiFAST Probe Hi-ROX Kit (Bioline, UK) according to the instruction of the manufacturer. Briefly, 50 ng of cDNA in a final volume of 10 µL was amplified using 0.5 µL of Taqman assay and 5 µL of SensiFAST Probe Hi-ROX mix. Taqman assays were used for *Gapdh* (Mm9999915_g1), *Cd36* (Mm00432403_m1), *Olr1* (Mm00454586_m1), *Ldlr* (Mm01177349_m1), *Scarb1* (Mm00450234_m1), *Cyp3a11* (Mm00731567_m1), *Cyp4a14* (Mm00484132_m1), *Pparg* (Mm01184322_m1), *Ppara* (Mm00440934_m1), *Lxr* (Mm00437265_g1), *Car* (Mm01283980_g1), *Hnf4a* (Mm01247712_m1) (ThermoFisher, USA). The fold changes of mRNA were determined using 2^−ΔΔCt^ method, using *Gapdh* as an internal control.

### Separation of murine blood plasma EVs

EVs were separated from plasma of control (normal diet) and HFD mice. The blood samples were collected from the inferior vena cava in tubes containing Acid Citrate Dextrose solution A (ACD A, Greiner Bio-One, Hungary), after performing euthanasia of the mice with CO_2_. The ACD tubes were used to prevent in vitro vesiculation [20]. The blood samples were spun once at 1500×*g* for 15 min [21]. Based on the protein and particle content, protein/particle ratio, EV and non-EV markers, we found that the number of centrifugations in plasma isolation protocol did not affect the EV content (Supplementary Fig. 1). Plasma samples were snap frozen and stored at − 80 °C. EVs were separated by differential centrifugation combined with size exclusion chromatography (SEC, qEV Original/70 nm, IZON, New-Zealand). The thawn up samples were diluted twofold with 0.2 µm filtered phosphate buffer saline (PBS). The samples were centrifuged at 2000×*g*, 4 °C for 30 min. The mEV fraction was separated at 12,500×*g*, 4 °C for 40 min. The supernatant was centrifuged at 100,000×*g*, 4 °C for 70 min to pellet sEVs. EV-containing pellets were resuspended in 500 µL 0.2 µm filtered PBS and were further purified with SEC. After rinsing the column with PBS, samples were applied onto the column and were fractionated according to the manufacturer’s protocol: a pooled EV fraction of 1500 µL was collected and the presence of EVs was confirmed both by flow cytometry and nanoparticle tracking analysis (NTA, ZetaView, ParticleMetrix GmbH, Germany). EVs were aliquoted, snap frozen and stored at − 80 °C until use. The detailed parameters of the centrifugation steps are summarized in Supplementary Table 2. The presence of EVs in the preparation was confirmed by transmission electron microscopy (TEM, Supplementary Fig. 2).

### Isolation of primary liver cells

Mice were anesthetized with a mixture of ketamine-xylazine (12–1.6 mg/mL, Medicus Partner Kft., Hungary). Liver cells were isolated by anterograde two-step collagenase perfusion according to F. Cabral et al., 2018, with minor modifications [22]. Briefly, pre-warmed (42 °C, Precision GP05 Wather Bath, ThermoFisher, USA), oxygen-enriched (VisionAire5 oxygen concentrator, AirSep Corporation, USA) solutions were used at a flow rate of 4 mL/min (EP-1 Econo pump, BioRad, USA). Desmosomes were disrupted with solution A (PBS + 0.5 M EGTA). Subsequently, the connective tissue of the liver was digested with solution B [3.9 g/L NaCl, 0.5 g/L KCl, 0.7 g/L CaCl_2_, 50 mM HEPES (ThermoFisher, USA) and collagenase IV (0.3 mg/mL, Worthington, USA)]. After the perfusion and subsequent excision of the liver, it was disrupted mechanically, and the tissue remnants were removed with a 100 µm filter (Miltenyi Biotec, Germany).

### Hepatocyte monoculture

Separated liver cells were processed as follows. HEPs were pelleted by low-speed centrifugation (50 g, 3 min, 4 °C), the viable cells were isolated with a 40% Percoll solution (Sigma, USA, 150 g, 7 min, 4 °C). The cells were cultured in Type I collagen-coated plates (15 µg/cm^2^, Sigma, USA) and were seeded in either 24-well plates for EV uptake studies (1.5 × 10^5^ cells/well) or in 6-well plates for EV production studies (10 × 10^5^ cells/well) in a seeding medium (DMEM (high glucose, 4.5 g/L), 10% fetal bovine serum (FBS, Gibco), 1000 U/L penicillin, 1000 µg/L streptomycin, 2 mmol/L l-glutamine, 7.5 mg/L Hydrocortisone, 0.02 mg/L epidermal growth factor, 0.014 mg/L glucagon, 1 mM sodium pyruvate, 0.5 ml/L insulin (all from Sigma, USA)). The cells were allowed to attach to the plate surface for 2–3 h at 37 °C and 5% CO_2_. After the incubation, the medium was changed to a FBS-free seeding medium.

### Fatty acid treatment

We simulated hyperlipidemic conditions in vitro by exposing cells to free fatty acids (FFA) for 16 h. Oleic acid (OA) and palmitic acid (PA) were dissolved in DMEM (high glucose, 4.5 g/L) containing 10% bovine serum albumin (BSA, Sigma, USA) with overnight rotation (10 rpm, Fisherbrand™ MiniTube Rotator, ThermoFisher, USA). Stock solutions of FFA were further diluted in FBS-free seeding medium to a final concentration of 400 µM OA and 200 µM PA.

### Assessment of the production of albumin and uric acid

Albumin production of HEPs was measured using a Mouse Albumin ELISA Kit (Abcam, UK). Uric acid concentration in the conditioned medium was analyzed using Amplex™ Red Uric Acid/Uricase Assay Kit (ThermoFisher, USA).

### Separation of hepatocyte-derived EVs

EVs were separated from serum-free conditioned medium of HEPs collected 16 h after HEPs isolation from 5 × 10^6^ control and 5 × 10^6^ FFA-treated cells. The protocol used for plasma EV separation was followed for hepatocyte-derived EV separation with minor modifications. Briefly, before the 2000×*g* centrifugation step, the conditioned medium was centrifuged at 300×*g*, 20 °C for 10 min and filtered (5 µm, Millipore, USA) to remove cells. The detailed parameters of the centrifugation steps are summarized in Supplementary Table 3. The presence of EVs was confirmed by TEM (Supplementary Fig. 3).

### Separation of HEK293T-palmGFP-derived EVs

HEK293T-palmGFP cell line (generous gift of Xandra Breakefield and Charles Lai) was grown in DMEM (low glucose, 1 g/L) medium, supplemented with 10% FBS (Gibco, USA), 1000 U/L penicillin, 1000 µg/L streptomycin, 2 mmol/L l-glutamine (Sigma, USA). Cells were maintained at 37 °C in a humidified atmosphere containing 5% CO_2_. Conditioned medium (160 mL) was collected 24 h after culture in serum-free medium in 8 T-175 TC treated flasks (Eppendorf, Germany). The passage number of cells ranged from 8 to 18, reaching 70–80% confluence, representing 131.7 ± 42.8 × 10^6^ cells per separation, with a viability of 90.1 ± 2.0%. Flow cytometric analysis of apoptosis in HEK293T-palmGFP cells is shown in Supplementary Fig. 4. The ratio of total apoptotic cells was below 7%. Cell cultures were regularly monitored for Mycoplasma. The conditioned medium was centrifuged at 300×*g*, 20 °C for 10 min and filtered (5 µm, Millipore, USA) to remove cells. The supernatant was centrifuged at 2000×*g*, 4 °C for 30 min to pellet lEVs. The mEVs were then separated at 12,500×*g*, 4 °C for 40 min. The supernatant was concentrated and further purified using tangential flow filtration (TFF-Easy, 20 nm pore size, HansaBioMed Estonia) and was centrifuged at 100,000×*g*, 4 °C for 70 min to pellet sEVs. After separation, the EV fractions were re-suspended in 0.2 µm filtered PBS, aliquoted, were snap frozen and were stored at − 80 °C until use. The detailed parameters of the centrifugation steps are summarized in Supplementary Table 4.

### Hepatocyte: non-parenchymal liver cell (NPC) co-culture

Hepatocytes were isolated as described above. Additional liver cell populations were isolated as published earlier, with minor modifications [23, 24]. Briefly, isolated HEPs were allowed to attach to a 24-well plate for 1 h at 37 °C, 5% CO_2_, then collagen Type I was overlaid on the cells (15 µg/cm^2^, Sigma, USA). The collagen was allowed to form a gel for 45 min, while NPCs were isolated from the HEP supernatant by differential centrifugation (300×*g*, 10 min, 4 °C). The viable cells were isolated with 33% Percoll solution (700×*g*, 12 min, 4 °C) and live HEPs were removed by centrifugation (1. 50×*g*, 3 min, 4 °C; 2. 70×*g*, 3 min, 4 °C). NPCs were seeded on HEPs at a density of 1 × 10^5^ cells/well in FBS-free seeding medium. After 2 h of incubation, the medium was changed and non-adherent cells were removed.

### Oil red O staining

Triglyceride accumulation of liver cells was visualized by Oil Red O (Sigma, USA) staining. Cells were fixed with 4% paraformaldehyde (PFA) in PBS, washed with PBS and stained with 60% Oil Red O diluted with distilled water. After washing with PBS and distilled water, cells were examined with a Nikon Diaphot TMD Inverted Microscope (Nikon, Japan).

### Immunocytochemistry

Cells were grown on coverslips (12 mm, VWR, USA) for 16 h after isolation, were washed with PBS and fixed with 4% PFA. Then, samples were blocked with PBS containing 5% FBS, supplemented with 0.3% Triton X-100 (Molar Chemicals Kft., Hungary) for intracellular albumin labeling. HEPs (albumin positive), KCs (F4/80 positive), and LSECs (CD146 positive) were identified in a co-culture by antibodies to cell-specific markers by immunocytochemistry. Primary antibodies were incubated overnight at 4 °C with the indicated dilutions (rabbit polyclonal anti-human serum albumin, 1:100; mouse monoclonal anti-CD146, 1:100; rat monoclonal anti-F4/80-eF660, 1:100). After washing out the primary antibodies, the secondary antibodies were incubated for 2 h at 37 °C with shaking (goat polyclonal anti-rabbit IgG-AF700, 1:100; goat polyclonal anti-mouse IgG-eF570, 1:100). Samples were washed with PBS, distilled water and covered with ProLongTM Gold antifade reagent with DAPI (ThermoFisher, USA). The slides were examined with Leica TCS SP8 Confocal Laser Scanning Microscope (Leica, Germany). All antibodies used in these experiments are listed in Supplementary Table 1.

### Cytokine secretion measurements

Cytokine production of liver cells was measured from the conditioned medium using a LEGENDplex™ Mouse Macrophage/Microglia Panel 13-plex (BioLegend, USA) and CytoFLEX S flow cytometer (Beckman Coulter, USA) according to the instruction of the manufacturer.

### Transmission electron microscopy of EVs

The morphology of EVs was examined by TEM. Samples were prepared according to Théry et al. 2006 with minor modifications [25]. Briefly, 2 µL of a sample was placed onto the surface of a formvar-coated grid, and was incubated for 10 min at RT. The residual solution was removed and fixed with 2% glutaraldehyde for 10 min. The grids were washed three times for 5 min, and we used uranyl oxalate for 10 min for contrast enhancement. The samples were further contrasted and embedded in a mixture of 4% uranyl acetate and 2% methyl cellulose and were examined by JEOL 1011 transmission electron microscope (Japan). HEK293T-palmGFP-derived mEVs were also visualized using a different approach. Transmission electron microscopy of ultrathin sections of EV pellets was carried out (Supplementary Fig. 5). In this case, the mEVs of the 12.5 k pellet were processed as described previously [26].

### Nanoparticle tracking analysis (NTA) of EVs

The size distribution of EVs was analyzed by NTA. The measurements were performed on a ZetaView PMX-120 instrument using ZetaVIEW software. The detailed parameters of the measurements are summarized in Supplementary Table 5.

### Protein and lipid assays of EVs

The protein concentration was measured by Micro BCA assay (ThermoFisher, USA). The lipid content was assessed by the optimized sulfo-phospho-vanillin (SPV) lipid assay according to Visnovitz et al., 2019 [27]. The lipid content of EVs was used as a basis of normalization in our EV uptake studies.

### Flow cytometry of EVs

EV-associated markers were analyzed by flow cytometry (CytoFLEX S, Beckman Coulter, USA). Both sEVs and mEVs secreted by HEK293T-palmGFP cells were bound onto the surface of latex beads (3 µm, 4% w/v Aldehyde/Sulfate Latex Beads, ThermoFisher, USA) overnight at 4 °C. One µg lipid/sample sEVs (~ 8 × 10^9^ particles) and mEVs (~ 4 × 10^8^ particles) were bound onto the beads. In the case of plasma and HEP-derived EVs, identical volumes (200 µL) of the 1500 µL pooled SEC fraction were co-incubated with the beads. Afterwards, the beads were blocked with 100 mM glycine and 2.5% w/v BSA solution. HEK293T-palmGFP EVs were stained with AxV-AF647 (1:100), mouse anti-human monoclonal CD63-PerCP (1:100), mouse anti-human monoclonal CD81-PerCP/Cy5.5 (1:100), mouse anti-human monoclonal CD9-APC (1:100), mouse anti-human monoclonal ApoB (1:100) labeled with goat anti-mouse polyclonal IgG-eF570 (as detailed in Supplementary Table 1).

Plasma/HEP EVs were stained with AxV-Pacific Blue (1:100), rat anti-mouse monoclonal CD63-APC (1:200), hamster anti-mouse monoclonal CD81-PE (1:400), mouse anti-human monoclonal ApoB (1:100) labeled with goat anti-mouse polyclonal IgG-eF570 (as detailed in Supplementary Table 1). Five thousand events/sample were measured by flow cytometry. Data were analyzed using FlowJo (Becton Dickinson, USA). In addition to the % positivity of the beads for the selected markers, the relative mean fluorescence intensity (RMFI) was also determined, which may provide additional information on the number of particles associated with a single bead [28].

### Flow cytometry analysis of EV uptake

In the in vitro experiments, liver cells were detached from the plate surface with Accutase solution (Sigma, USA) and were analyzed directly after a centrifugation step (50×*g*, 3 min at 4 °C). NPC cells were separated from HEPs by centrifugation. The pellet was re-suspended in a special buffer to provide optimal conditions for liver cell survival and subsequent immunostaining (1:20 dilution of MACS BSA Stock Solution with autoMACS Rinsing Solution, Miltenyi Biotec, Germany) containing FcR Blocking Reagent (1:10). After 10 min incubation, the excess reagent was removed by centrifugation, and the cells were re-suspended in solution (rat anti-mouse monoclonal CD146-PerCP-Vio700, 1:50; recombinant human anti-mouse monoclonal F4/80-PE, 1:100 in MACS buffer). After washing out the antibodies, the cells were stained with TO-PRO-3 (1:3000) and measured by flow cytometry. Within the population of living cells, CD146^+^ (LSECs) and CD146^−^ cells were gated. F4/80^+^ (KCs) cells were then identified within the CD146^−^ population according to Lynch et al., 2018 (Supplementary Fig. 6) [29]. All antibodies and viability dye used in these experiments are listed in Supplementary Table 1.

### Detection of EV fluorescence by a fluorescence-labeled organism bioimaging instrument

The GFP positivity of the HEK293T-palmGFP-derived EVs was demonstrated with Fluorescence-labeled Organism Bioimaging Instrument (FOBI, Neoscience, Suwon City, Rep. Korea) under certain parameters (“Blue channel” light source (455 nm) and 520 nm long pass emission filter for detection). The exposure time was 1000 ms/image with gain level 1. After sample preparation, the fluorescence of the HEK293T-palmGFP-derived sEVs (1 µg lipid in 28 µL), mEVs (1 µg lipid in 12 µL), and PBS as control sample were measured in Eppendorf tubes. Prior to every fluorescence scan, background image was registered in the same position using white light illumination (400–800 nm) without applying any filter for detection. In these cases, the exposure time was 100 ms with gain level 1. For image analysis, fluorescence images were co-registered to background scans by VivoQuant software (inviCRO, Boston, US).

### Radiolabeling of HEK293T-palmGFP-derived EVs and in vivo SPECT/CT imaging

Radiolabeling of HEK293T-palmGFP-derived sEV and mEVs was performed with the ^99m^Tc-HYNIC-Duramycin kit (Molecular Targeting Technologies, Inc, U.S.A.). In the first step, HYNIC-Duramycin was labeled with ^99m^Tc according to the manufacturer’s instructions. 3.5 GBq [^99m^TcO4]^−^ was eluted in 0.5 mL saline and added to the kit, followed by placing the vial at 80 °C for 20 min. Radiochemical yield of this step was checked with reversed-phase HPLC [30], and found to be greater than 95%. Next, 900 MBq ^99m^Tc-HYNIC-Duramycin in 200 µL was added to 60 µL sEV and mEV samples (3 µg total lipid in each) and diluted with 240 µL PBS and incubated at 30 °C for 20 min with shaking at 200 rpm. Purification of the labeled EVs was performed by SEC using a *V*_T_ = 3.5 mL column filled with Sepharose CL-2B gel (GE Healthcare Bio-Sciences AB, Sweden). 0.5 mL sample was pipetted on the column, and 0.5 mL fractions were collected using PBS as eluent. The fraction corresponding to the void volume of the column (from 1 to 1.5 mL elution volume) contained the radiolabelled EVs. Radiochemcial purity was checked by radio-HPLC-SEC using a Tricorn 5/100 glass columns (GE Healthcare Bio-Sciences AB, Sweden), filled with Sepharose CL-2B [31].

In vivo imaging was performed with a NanoSPECT/CT Silver Upgrade (Mediso Ltd, Hungary) sequential animal SPECT/CT imaging system. In the SPECT/CT experiment, 12 ± 3 MBq of ^99m^Tc-HYNIC-Duramycin labeled sEV and mEV samples in 100 µL volume was injected into the tail vein of C57BL/6 mice (*n* = 2 for both sEV and mEV samples). During the scans, the animals were continuously anaesthetized using a mixture of 1–1.5% isoflurane and medical oxygen, and their body temperature was maintained at 37 °C throughout the scanning. CT and subsequent SPECT imaging started 1 h after the time of administration, and the acquisition lasted 10.5 and 30 min, respectively. Reconstructed images were analyzed with Fusion (Mediso Ltd, Hungary) and VivoQuant (inviCRO LLC, US) image analysis software by placing appropriate Volume of Interests (VOI) manually on the organs in each CT scans. Radioactivity concentrations in MBq/cm^3^ were determined for each VOI and corrected for scattering and isotopic decay in the reconstruction algorithm. The uptake values were measured in the heart, lungs, kidneys, bladder, liver, spleen and bones.

### Statistical analysis and graphic software

Values are reported as mean ± standard deviation (SD). Statistical analyses were performed using GraphPad Prism 7.00 (USA). The normal distribution of the data was examined using the Shapiro–Wilk normality test. For normally distributed data, unpaired/paired *T* test or one-way/two-way ANOVA test was performed. Post hoc analyses of significance were made by Tukey’s multiple comparisons test. If the data did not pass the normality test, a Mann–Whitney test was performed. *p* < 0.05 was accepted as statistically significant. The figures were generated using GraphPad Prism 7.00 (USA).

## Results

###  Characterisctics of HFD-fed mice

**Fig. 1:**
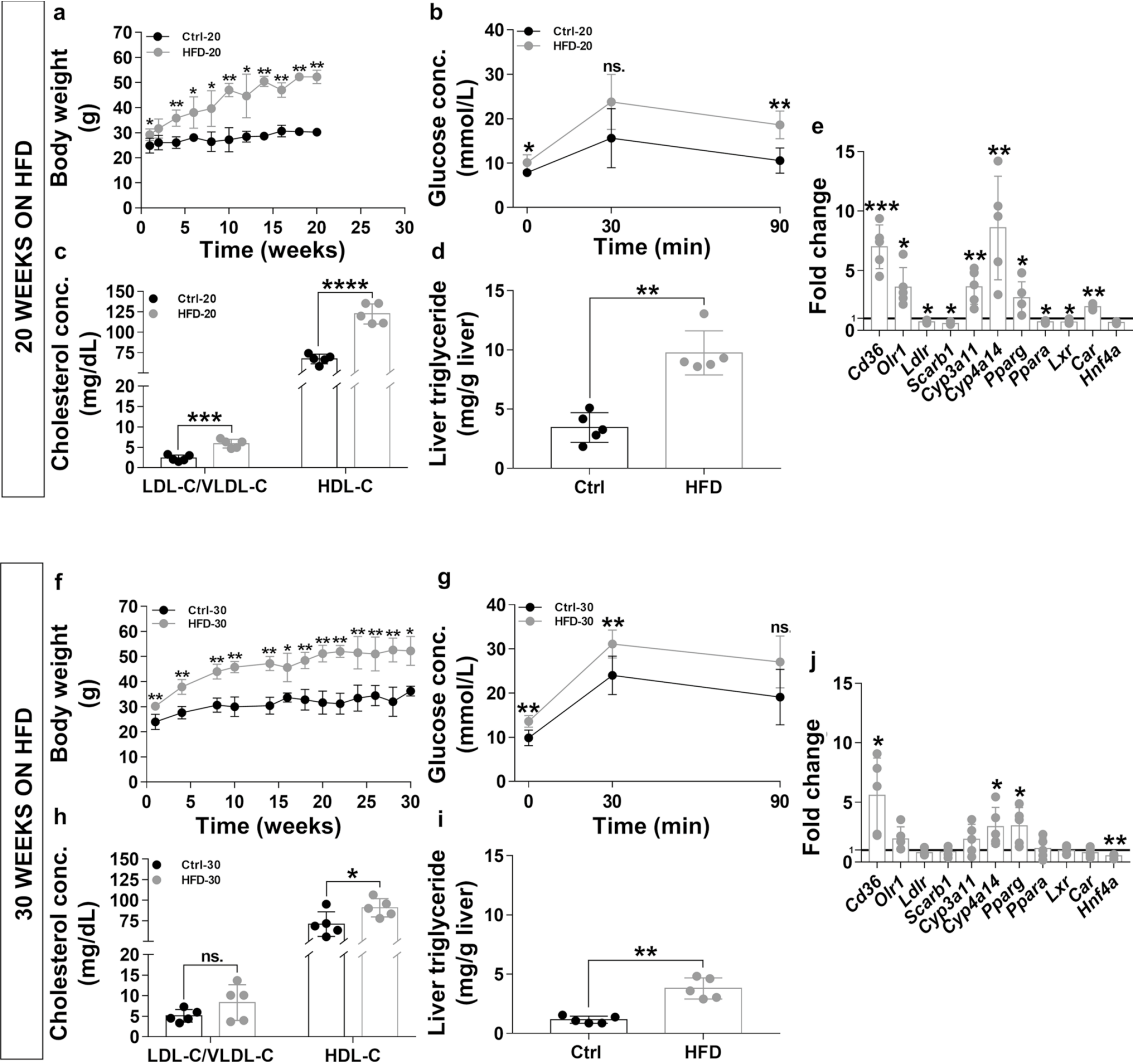
Effect of HFD on body weight, glucose tolerance, plasma HDL-C and LDL-C/VLDL-C levels, liver TG concentration and mRNA expression levels. The effect of HFD on body weight was monitored regularly (**a** and **f**). After 20 and 30 weeks of HFD, the fasting glucose levels and glucose tolerance were investigated (**b** and **g**). The development of hyperlipidemia was confirmed by the HDL-C and LDL-C/VLDL-C measurements (**c** and **h**). We examined the accumulation of hepatic TG in response to hyperlipidemia (**d** and **i**). We also investigated changes in liver mRNA expression using RT-qPCR (**e** and **j**). Values are reported as mean ± SD. *p* values are calculated by Mann–Whitney test (**a** and **f**) or unpaired *T* test (**b**, **c**, **d**, **e**, **g**, **h**, **i** and **j**). *n* = 5 was in each group. *ns* not-significant, * < 0.05, ** < 0.01, *** < 0.001, **** < 0.0001

HFD increases the risk to develop metabolic disorder [32]. Here, we found a significant increase in the body weight even after 1 week on HFD (Fig. 1a, f). The mean body weight increased by 17.7 ± 9.2% and 26.2 ± 5.9% in the HFD-20 and HFD-30 groups, respectively. Continued weight gain was observed at 20 and 30 weeks (by 72.8 ± 8.9% and 42.0 ± 15.8%, respectively). The HFD also affected blood sugar levels (Fig. 1b, g). The blood glucose levels in the HFD groups were significantly higher after 6 h fasting than in the control groups. Following intraperitoneal glucose injection, blood glucose levels in the HFD groups remained elevated during the time of the experiment (90 min). To determine dyslipidemia, plasma LDL-C/VLDL-C and HDL-C levels were measured (Fig. 1c, h). Compared to the control groups, the HFD significantly increased plasma LDL-C/VLDL-C and HDL-C levels. In hyperlipidemia, liver accumulates triglycerides (TG) that can lead to fatty liver (Fig. 1d, i). Liver TG levels were significantly higher in both the HFD-20 and the HFD-30 groups. The expression of genes important in the pathogenesis of fatty liver was examined by RT-qPCR (Fig. 1e, j). The gene expression of many receptors involved in the uptake of lipoproteins and FFAs were altered. In the HFD-20 group, there was a significant increase in the *Cd36*, *Olr1* and a decrease in *Ldlr* and *Scarb1* expression. Furthermore, the mRNA levels of *Cyp3a11 and Cyp4a14* enzymes associated with fatty liver development were increased. The nuclear receptors, implicated in the metabolism and storage of FFAs showed the following pattern: *Pparg*, *Car* mRNA levels were increased, while *Ppara* and *Lxr* were decreased. In the HFD-30 group, only the *Cd36*, *Cyp4a14* and *Pparg* expression was upregulated. In contrast, the expression of *Hnf4a* was significantly reduced.

### Effect of hyperlipidemia on the plasma EV content

**Fig. 2:**
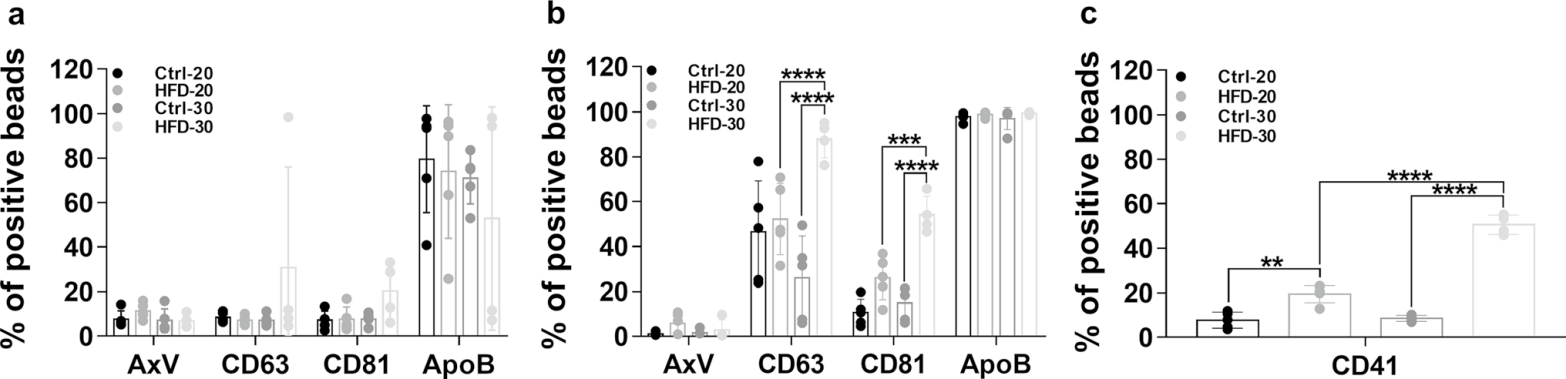
Flow cytometric characterization of plasma-derived EVs based on their EV-specific marker expression. The effect of HFD on plasma EV content was determined by EV marker expression. EVs were purified by SEC and 200 µL of the pooled 1500 µL EV-containing elutes were bound onto latex beads. HFD did not affect the plasma mEV content (**a**). Bead-bound plasma sEVs resulted in a significantly increased CD63, CD81 and CD41 positive bead frequency in the HFD-30 group as compared to the Ctrl-30 and HFD-20 groups (**b** and **c**). Values are reported as mean ± SD. *p* values were calculated by two-way ANOVA (**a** and **b**) or one-way ANOVA (**c**) and Tukey’s multiple comparisons post hoc test. *n*_Ctrl-20_ = 5, *n*_HFD-20_ = 5, *n*_Ctrl-30_ = 5, *n*_HFD-30_ = 4 (one plasma sample was excluded due to significant hemolysis), ** < 0.01**,** *** < 0.001, **** < 0.0001

Next, we investigated the effect of HFD on the concentration of circulating plasma EVs. Based on the analysis of EV markers, there was no change in the plasma mEV content (Fig. 2a, Supplementary Fig. 7a). ApoB^+^, mEV-coated beads were detected at a strikingly higher frequency and higher RMFI value as compared to AxV, CD63 and CD81 positive ones. The staining pattern of bead-bound sEVs was different from what we have seen in the case of mEVs. While AxV^+^ beads were present at low frequency, we found high frequency for CD63, CD81 and ApoB (Fig. 2b) sEVs compared to mEVs. The proportions of CD63^+^ and CD81^+^ sEVs were significantly increased in the HFD-30 group (based on the number of the positively stained beads). However, there was no significant difference in CD63, CD81 RMFI values (Supplementary Fig. 7b). ApoB positivity was tested, because ApoB is known to co-isolate with EVs [33]. Interestingly, ApoB^+^ particles/ApoB molecules were present on 100% of the beads, regardless of group (Fig. 2b). In contrast, the RMFI value of ApoB was significantly higher in the HFD-20 and HFD-30 groups (Supplementary Fig. 7b), which correlates well with plasma LDL-C/VLDL-C concentration. We tested CD41^+^ EVs, because it is known that CD41^+^ platelet-derived EVs can induce platelet aggregation, and thus, may contribute to the development of cardiovascular diseases [34, 35]. It can be clearly seen that HFD significantly increases the percentage of CD41^+^ beads after 20 and 30 weeks (Fig. 2c). Additionally, the RMFI value of CD41 was significantly higher in the HFD-30 group (Supplementary Fig. 7c). The results of the antibody control experiments are shown in the Supplementary Table 6. We were also interested if there was any difference in the size distribution of EVs in the blood plasma of mice kept of HFD for 20 or 30 weeks. Based on our NTA measurements, we found no difference in the size distribution of EVs. The median size (X50), particle concentration and protein/particle number ratio of the EVs are shown in the Supplementary Table 7.

### Characteristics of liver cell cultures

**Fig. 3:**
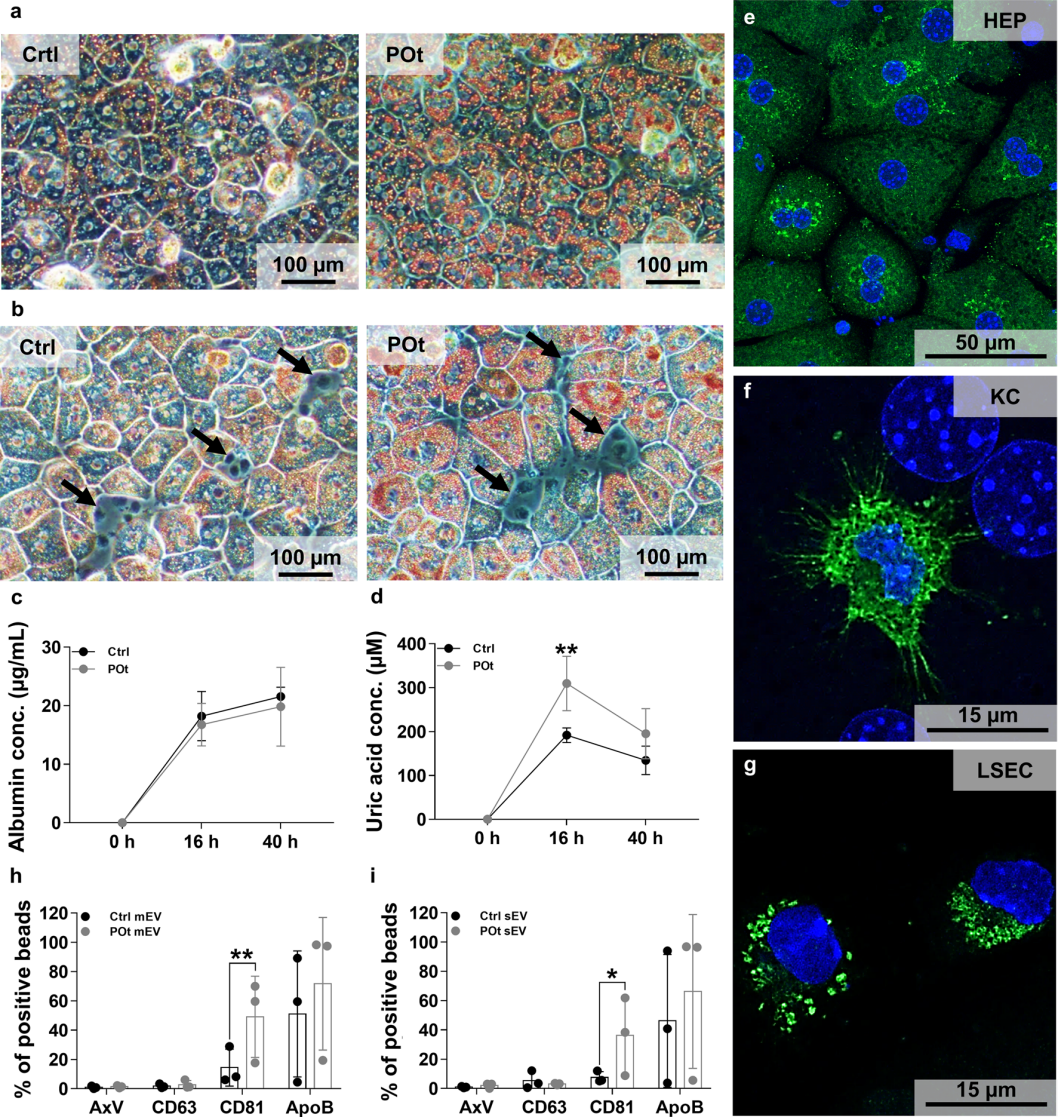
Characterization of liver cell cultures and hepatocyte-derived EVs. In vitro*,* hyperlipidemic conditions were modeled by FFA treatment. Triglyceride accumulation in HEP monoculture (**a**) and HEP-NPC co-culture (**b**) was demonstrated by Oil Red O staining. While no lipid accumulation was seen in NPCs (black arrows), there was a high number of lipid droplets in HEPs. HEPs rapidly de-differentiate in vitro, leading to decreased albumin production. During our experiments, albumin production remained unchanged (**c**). The production of uric acid as a marker of hyperlipidemia was significantly increased after FFA treatment (**d**). HEPs were identified in HEP-NPC co-culture by their albumin production (**e**), KCs by F4/80 immunostaining (**f**), and LSECs by their CD146 cell surface expression (**g**). Low power magnification microphotographs are shown in Supplementary Fig. 12. The effect of POt on HEP-derived EVs was determined by EV marker expression. EVs were purified by SEC and 200 µL of the 1500 µL EV-containing elutes were bound to latex beads. HEP-derived CD81^+^ mEVs and sEVs carrying bead frequencies were elevated in the POt group (**h** and **i**). Values are reported as mean ± SD. p values were calculated by two-way ANOVA and Tukey’s multiple comparisons post-hoc test. *n*_Ctrl_ = 3, *n*_POt_ = 3, * < 0.05, ** < 0.01

Primary liver cells were cultured either in HEP monoculture or in HEP-NPC co-culture. Hyperlipidemic conditions were in vitro simulated with the mixture of oleic (400 µM) and palmitic (200 µM) acids. As shown in Fig. 3a (left panel), cuboidal HEPs were grown in regular FBS-free seeding medium. They contained accumulated lipid droplets in their cytoplasms as identified by Oil Red O staining. Of note, the size of the lipid droplets in HEPs increased markedly after 16 h FFA treatment (Supplementary Fig. 8), while NPC cells apparently did not accumulate fat droplets (Fig. 3b, black arrows). It is known that in vitro conditions may lead to de-differentiation of HEPs leading to a decrease in specific cell functions [36]. Albumin production is considered as an important indicator of HEP function. The secreted albumin remained unchanged in control and palmitic acid/oleic acid treated (POt) culture during the course of the experiments (Fig. 3c). Uric acid production correlated positively with hyperlipidemia (as a marker of hepatic steatosis). It can be clearly seen that after 16 h FFA treatment, there is a significant increase in the uric acid production by HEPs (Fig. 3d). The liver cell co-culture contained HEP:LSEC:KC:other cells in 6:2:1:1 ratios (which is the case for normal mouse liver). HEPs (1.5 × 10^5^) were cultured with 1 × 10^5^ NPCs. The ratio of KCs to LSECs was validated by flow cytometry and was found 22.9 ± 4.8%: 39.9 ± 4.8% (Supplementary Fig. 9). The presence of HEPs (identified by intracellular albumin immunostaining), KCs (detected by F4/80 positivity), and LSECs (recognized by CD146 expression) in the co-culture was confirmed (Fig. 3e, f, g). Different liver cell types were reported to secrete various cytokines [37, 38]. In line with this, testing our liver cell cultures, we also found evidence for the secretion of numerous cytokines (Supplementary Fig. 10). This cytokine environment was maintained even under hyperlipidemic conditions, where only the production of CXCL1 in HEP monoculture and the production of CCL22, GCSF, CXCL1 in HEP-NPC co-culture were significantly increased. However, we did not detect a pronounced pro-inflammatory cytokine response.

### Effect of hyperlipidemic conditions on the release of hepatocyte-derived EVs

Next, we studied the effect of hyperlipidemia on the EV production by HEPs in vitro. EVs were separated from serum free conditioned medium of control and POt cells. The proportion of both AxV^+^ and CD63^+^ HEP-derived mEVs and sEVs were low, and was unaffected by FFA treatment. In contrast, POt significantly increased the proportion of both CD81^+^ mEVs (Fig. 3h) and sEVs (Fig. 3i). There was no statistically significant change in RMFI values of CD81 (Supplementary Fig. 7d and e). The results of the antibody control experiments are shown in the Supplementary Table 6. Similarly to what we have found in the case of circulating EVs, our NTA measurements did not reveal any difference in the size and distribution of EVs released by HEPs under control and POt conditions. The median size (X50), particle concentration and protein/particle number ratio of the EVs are shown in the Supplementary Table 8.

### Characterization of HEK293T-palmGFP-derived EVs

**Fig. 4:**
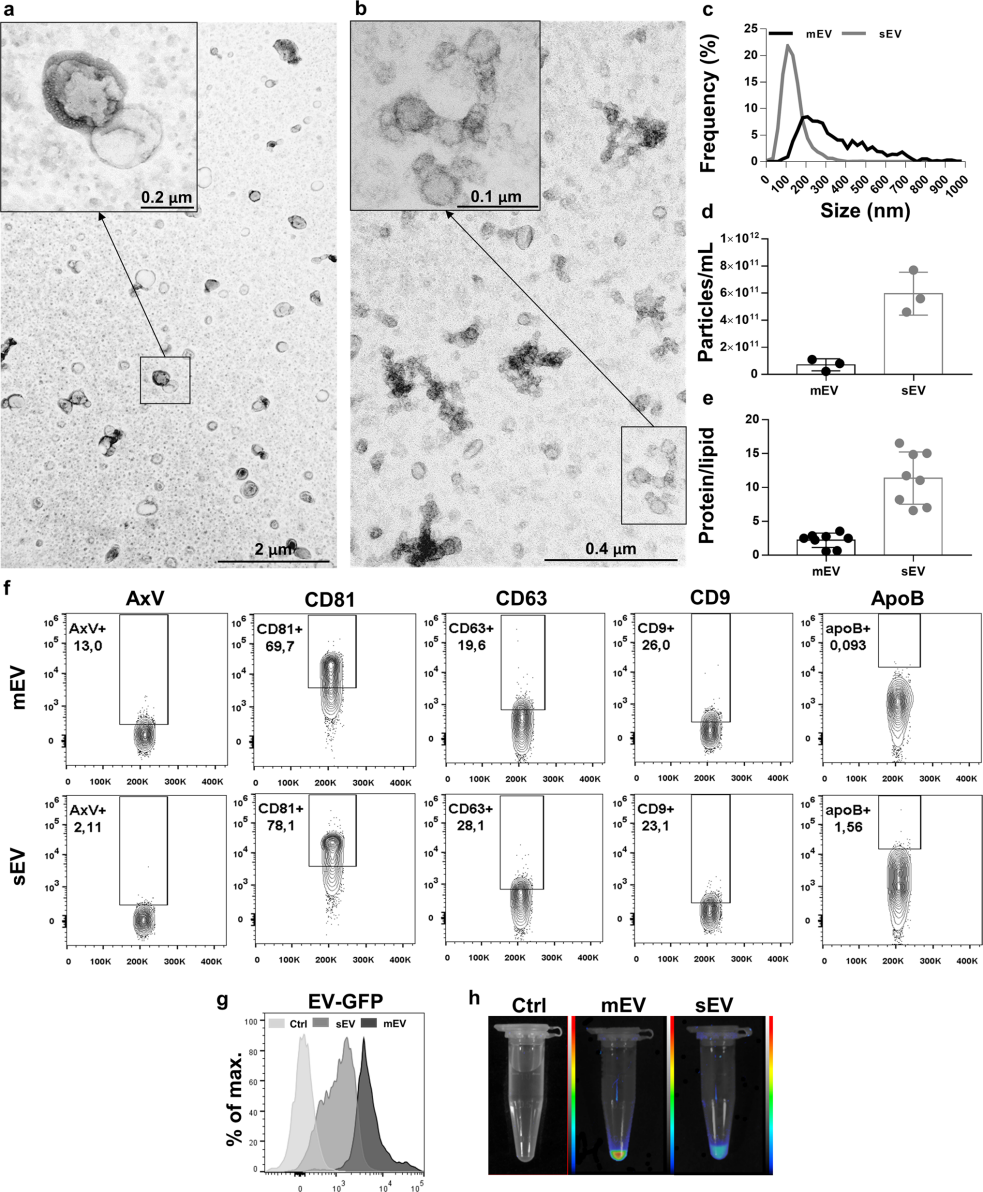
Characterization of HEK293T-palmGFP-derived EVs. The morphology of EVs was examined by TEM (**a** and **b**), the size distribution was determined by NTA (**c**). Size-based EV fractions were characterized by their particle concentration (**d**, *n* = 3) protein/lipid ratios (**e**, *n* = 8). The presence of HEK293T-palmGFP-derived EV-associated protein markers and the intrinsic fluorescence of EVs were analyzed by flow cytometry. Identical amounts (1 µg lipid containing) mEVs and sEVs were adsorbed onto the surface of latex beads prior to immunostaining (**f** and **g**). The intrinsic fluorescence of EVs was confirmed by FOBI (**h**) (intensity values min. 20 to max. 130). mEVs (black symbols), sEVs (grey symbols)

mEVs and sEVs were separated from the conditioned medium of HEK293T-palmGFP cell line and were characterized by TEM, NTA, protein and lipid content. The presence and characteristic morphology of EVs were demonstrated by TEM (Fig. 4a, b). Based on the NTA measurements (Fig. 4c and d), the median size (X50) of mEVs and sEVs were 326.3 ± 19.8 and 130.5 ± 5.8 nm, respectively. The protein/lipid ratio was found to be 2.2 ± 1.1 and 11.4 ± 3.9 for mEVs and sEVs, respectively (Fig. 4e). EV-associated markers were also identified using flow cytometry (Fig. 4f). We found a very prominent CD81 positivity of latex bead bound mEVs and sEVs. The results of the antibody control experiments are shown in Supplementary Fig. 11. The GFP fluorescence of EV fractions was shown by flow cytometry (Fig. 4g) and FOBI (Fig. 4h).

### In vivo biodistribution of HEK293T-palmGFP-derived EVs

**Fig. 5:**
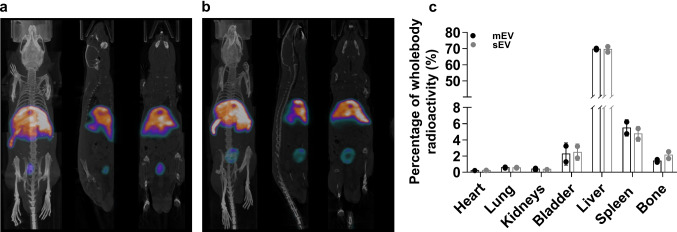
In vivo SPECT/CT images of the distribution of ^99m^Tc-HYNIC-Duramycin-labeled HEK293T-palmGFP sEV (**a**) and mEVs (**b**). The 3D reconstructed and co-registered SPECT and CT images are shown together with sagittal and coronal images (from left to right) (**a** and **b**). Quantitative distribution of sEV and mEV samples (**c**). Activities are measured in the specified organs as a percentage of the wholebody radioactivity

In our ^99m^Tc-labeling experiments, we obtained a label yield of 60 to 80 MBq/3 µg total lipid in 0.5 mL volume. As revealed by radio-HPLC-SEC, the purity of labeled EV samples was above 95%, which is sufficient for in vivo investigations. Figure 5a, b shows typical SPECT/CT images of the in vivo biodistribution of sEV and mEVs. For both samples, high accumulation of the injected EVs can be observed in liver and spleen in accordance with previous literature data [39]. Radioactivity in the bladder, which does not succeed 3% of the total injected activity, can be attributed to free ^99m^Tc-HYNIC-Duramycin. This observation also indicates an in vivo stability of the labeling, because free ^99m^Tc-HYNIC-Duramycin is quickly eliminated through the kidneys with a low uptake in the liver and intestines (22 ID%/g of the injected radioactivity can be detected in urine at 60 min after injection according to Zhao et al. [30]). Figure 5c shows the distribution of the labeled EVs within the different organs as a percentage of the whole body radioactivity. Liver uptakes of sEVs and mEVs were 69.5 ± 2.5% and 69.7 ± 0.7%, respectively. Spleen showed the second largest accumulation of radioactivity with 4.8 ± 1.0% and 5.5 ± 1.0%, for mEVs and sEVs, respectively. In summary, in vivo SPECT/CT imaging with ^99m^Tc-HYNIC-Duramycin revealed that the liver takes up the majority of intravenously injected EVs, and no significant difference can be observed in this regard between sEVs and mEVs.

### EV uptake in hepatocyte monoculture

**Fig. 6:**
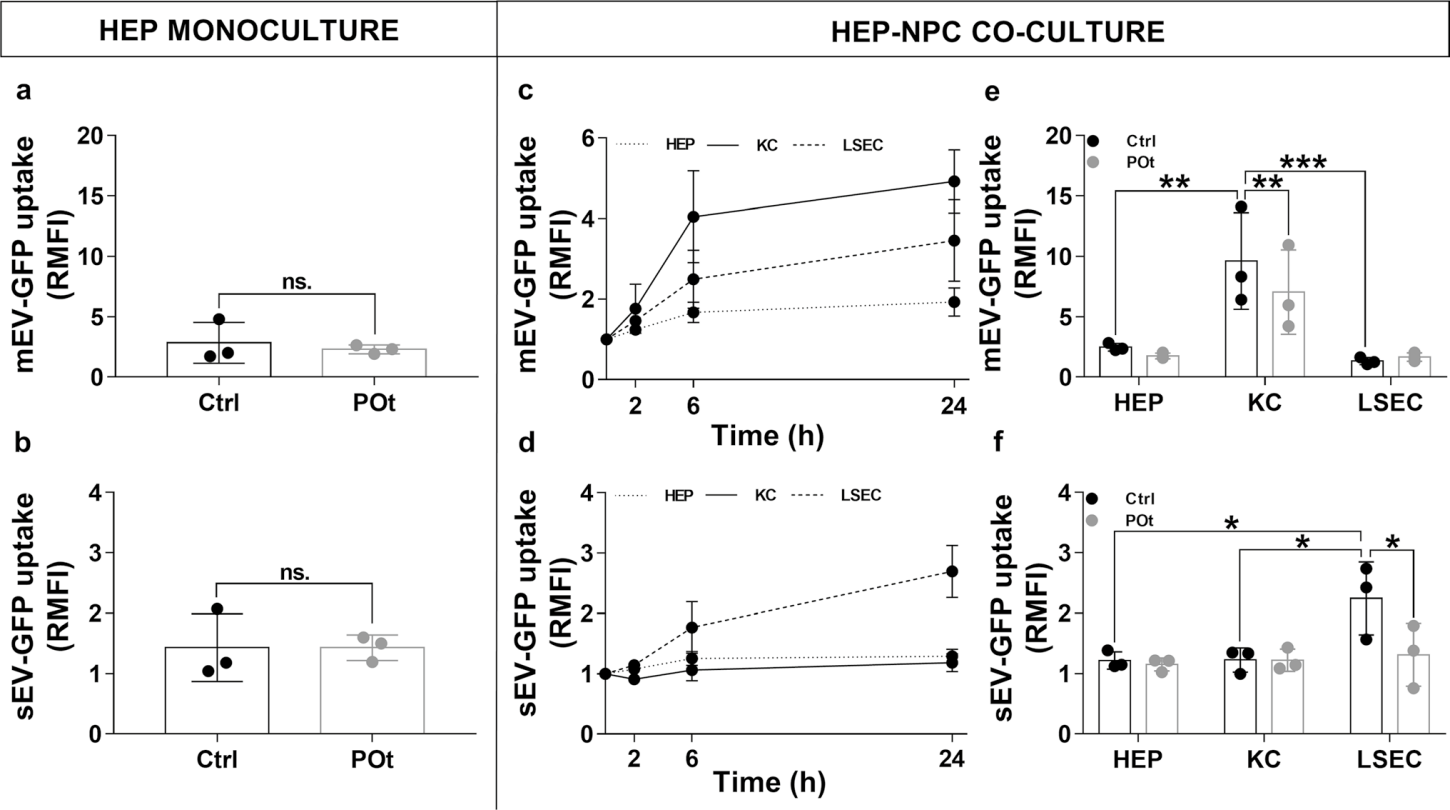
In vitro uptake of HEK293T-palmGFP-derived EVs by liver cells. The uptake of EVs was examined in HEP monoculture under control and POt conditions. HEPs were able to take up both mEVs (**a**) and sEVs (**b**) and the hyperlipidemic condition did not significantly affect the uptake of EVs. Based on our time course experiments (**c** and **d**), we tested the effect of hyperlipidemic conditions on EV uptake at 24 h in HEP-NPC co-culture. The mEV uptake of KCs (**e**) and sEV of LSECs (**f**) decreased significantly as a result of the FFA treatment. Values are reported as mean ± SD. *p* values were calculated either by paired *T* test (**a** and **b**) or by two-way ANOVA and Tukey’s multiple comparisons post-hoc test (**e** and **f**). *n*_Ctrl_ = 3, *n*_POt_ = 3, *ns* not-significant, *p < 0.05, **p < 0.01, ***p < 0.001

HEPs comprise about 80% of the total cells and volume of the liver [40]. The question arises as to what extent they are able to internalize and eliminate EVs. To answer this question, we investigated the uptake of HEK293T-palmGFP EVs in HEP monoculture. In preliminary experiments, we found that EVs were taken up by HEPs in a dose dependent manner, reaching a maximum at 24 h (data not shown). Figure 6a, b shows results obtained after incubation of 1 µg lipid containing EVs with HEPs for 24 h in serum free condition. As demonstrated in Fig. 6a, b, HEPs were able to take up both mEVs and sEVs. Hyperlipidemic conditions did not affect the uptake of mEVs or sEVs significantly.

### EV uptake in HEP-NPC co-culture

LSECs and KCs play a complementary role in elimination of macromolecules and particulates from blood circulation. To get a more precise picture of the role of the liver in the elimination of different nanoparticles, we examined the uptake of EVs in a HEP-NPC co-culture. EV uptake kinetics of HEPs, KCs and LSECs was examined in vitro (Fig. 6c, d). One μg lipid containing HEK293T-palmGFP mEVs or sEVs were added to liver cells and were incubated for 2, 6 and 24 h. After 6 h, we saw an increased EV uptake, which reached a maximum at 24 h (Fig. 6c, d). Interestingly, while mEVs were taken up primarily by KCs (Fig. 6e), the sEVs were internalized predominantly by LSECs (Fig. 6f). Under hyperlipidemic conditions, accumulation of lipid droplets in liver cells increased. To study EV uptake under hyperlipidemic conditions, cells were pre-treated with a mixture of palmitic acid-oleic acids. After 16 h of FFA pre-treatment, liver cells were incubated with mEVs or sEVs for 24 h. Our preliminary data showed that EV exposure shorter than 24 h did not affect the elimination of EVs. In contrast, we found under our in vitro hyperlipidemic model conditions significantly reduced mEV uptake by KCs and sEV by LSECs after 24 h EV exposure.

## Discussion

The goal of the present study was to examine the dynamics of EV release and uptake by liver cells. Importantly, here, we report for the first time the cell type-specific involvement of liver cells (including HEPs and the non-parenchymal KCs and LSECs) in the release and uptake of mEVs and sEVs. While mEVs were primarily taken up by KCs, sEV uptake was the highest in LSECs. Both mEV and sEV internalization was reduced under hyperlipidemic conditions.

The liver is considered to be a natural scavenger of EVs, independently of the species of origin of EVs. Wiklander and colleagues studied the biodistribution of EVs of different species origins [39]. They found that EVs from human (HEK293T) and rat (OLN-93) cell lines showed the same biodistribution pattern as compared to EVs from a mouse C1C12 cell line. Usually, the role of phagocytic innate immune cells is emphasized in EV elimination. Surprisingly, however, even after clodronate liposome-mediated depletion of macrophages, B16BL6-derived EVs still accumulated in the liver [12]. This suggests that cells other than macrophages contribute to EV elimination. Our in vitro experiments with HEP-NPC co-culture provide evidence that besides liver resident macrophages (KC), HEPs and LSECs are also able to take up EVs. KCs are known to have a key role in eliminating insoluble particles (≥ 100 nm) by phagocytosis, while LSECs are known to remove smaller particles (≤ 100 nm) from the circulation by pinocytosis [14]. In line with this, here, we found that presumably due to their size, mEVs were eliminated more efficiently by KCs and sEVs by LSECs.

Hyperlipidemia is a collective term for elevated levels of TGs, cholesterol, cholesterol esters and plasma lipoproteins [15]. Uptake of increased amounts of dietary fatty acids in the HFD lead to the absorption of enhanced amounts of FFAs from the lumen of the small intestine. Some FFAs are later liberated from the chylomicrons and are carried to tissues in an albumin-bound form. Chylomicrons later undergo numerous additional changes in their composition, and the so-called “chylomicron remnants” are eliminated from the circulation by the liver [41]. HFD is also known to induce insulin resistance [42], which in turn, also results in an increased level of FFAs.

In this work, we induced chronic hyperlipidemia by sustained exposure of mice to HFD (20–30 weeks), and the hyperlipidemic status was confirmed on the basis of elevated levels of both HDL-C and LDL-C/VLDL-C in the circulation of mice. In vitro, to mimic the hyperlipidemic conditions, we applied the commonly used POt model [43, 44]. Importantly, the same FFAs were predominant in both the HFD and in the FFA-containing tissue culture medium. The effect of diet-induced hyperlipidemia on plasma and HEP-derived EVs have been investigated previously [17, 18, 45–48]. In line with the previous findings, we detected elevated levels of circulating EV in mice kept on a chronic HFD. However, previous works examined mice after a shorter diet period, and they primarily focused on sEVs. Importantly, previous works only used differential centrifugation for EV separation [17, 18, 48]. Our data presented here demonstrate that secondary (non-genetic) hyperlipidemia is linked to both an increased circulating EV number and an elevated hepatic EV release. In addition to EVs, co-isolation of ApoB^+^ particles was also examined. Of the lipoproteins, chylomicrons, LDL, and VLDL particles carry ApoB on their surface. Plasma contains orders of magnitude more lipoproteins (10^16^) than EVs (10^9^), and HEPs secrete only VLDL particles [41, 49]. Although ApoB was indeed present in our samples, we would like to emphasize that we collected fasting plasma, therefore its chylomicron concentration was expected to be low according to our recently published data [33]. Moreover, in HEP cell cultures, one has to calculate only with VLDL and not with LDL or chylomicron. Besides potential co-isolation of LDL and VLDL particles, the ApoB positivity in our preparations could have resulted from the formation LDL and/or VLDL aggregates or EV-lipoprotein complexes (as we have shown earlier [33]). Most importantly, very recently, we have demonstrated the presence of a universal protein corona around EVs, viruses and synthetic lipid nanoparticles in blood plasma which also includes ApoB (in addition to ApoA1, ApoC3 and ApoE and other proteins) [50] suggesting that ApoB is not a co-isolated contamination but rather an organic part of the natural protein corona of EVs.

Here, we also report that liver cells under hyperlipidemic conditions had impaired uptake functions. Asanuma and colleagues showed with SPIO-MRI technology that rats and patients with NAFLD (a disease caused by hyperlipidemia) have reduced KC phagocytic function [19]. However, until our work, there has been no data on the possible interference of sEV uptake (~ 100 nm particles) with hyperlipidemia. Limitations of this study is the reported difference between the HDL-LDL balance in mice and in humans (mice lack of the cholesteryl ester transfer protein, an enzyme that transports cholesterol from HDL to LDL, [41]). However, in spite of the above limitations, our data highlight the potential association of EVs and hyperlipidemia.

While the EV field is expanding very rapidly and EVs are considered as very promising potential therapeutic vehicles and agents, rapid elimination of EVs from the circulation is a major limitation of the therapeutic application of EVs. The only exception being the category of liver diseases in the cases of which the natural hepatic docking of EVs eliminates the necessity of specific EV targeting. As a solution, local administration of EVs has been suggested in different studies [51–53]. A deeper understanding of the EV scavenger processes by individual liver cell populations may enable the development of therapeutic approaches with blocking or targeting different liver cell populations. Given that hyperlipidemia represents a major health problem in Western countries where reduced daily exercise and Westernized food leads to elevated levels of lipoproteins and FFAs in the circulation, the effect of hyperlipidemia on circulating EV levels may also have multiple implications. In studies on circulating EVs, not only the momentary dietary status (fasting versus postprandial) should be taken into account as we demonstrated previously [33], but also the fat content of the diet (e.g. that of a ketogenic diet) should be taken into account when considering baseline reference EV levels. In addition, elevated levels of circulating EVs under sustained hyperlipidemic conditions may have strong implication in different processes (e.g. in blood coagulation).

The ability to track and measure EVs biodistribution in vivo is essential for utilizing EVs as potential therapeutic vehicles. However, noninvasive methods have been lacking to determine biodistribution of EVs. Optical imaging for tracking EVs is hindered by limited tissue penetration of fluorescent and bioluminescent signals, as well as impractical quantitative analysis. Attempts to radiolabel EVs for SPECT and PET imaging are still in their infancy [7, 8, 54–57]. Duramycin is a phosphatidylethanolamine (PE)-binding peptide which was recently suggested for the radiolabeling of EVs (Clodagh O’Neill, Zhonglin Liu, Róisín Dwyer, Virtual ISEV workshop EV imaging in vivo September 10–11th, 2020). The rationale behind using duramycin for labeling of EVs is that it has high affinity and specificity to bind phosphatidylethanolamine (PE), an abundant phospholipid in EVs [30]. It has been believed that externalization of a significant portion of PE and phosphatidylserine (PS) from the inner to the outer leaflet of membrane is a key feature of EV formation. As a 19-amino-acid tetracyclic peptide, duramycin is featured with low molecular weight, highly stable structure, high binding affinity to the head group of PE, and higher binding percentage of EVs than cells. Our preliminary labeling results in this study and previous data acquired by Liu and his associates demonstrate that ^99m^Tc-labeled EVs with high yield and stability can be achieved via the specific duramycin-PE binding approach. Of note, the observed distribution in this study highly resembles that of ^99m^Tc-tricarbonyl-labeled erythrocyte-derived EVs [58]. Unlike ^99m^Tc-Duramycin binding to phospholipids of EV membrane, ^99m^Tc-tricarbonyl binds to several amino acids in membrane proteins, which may change the targeting features and biodistributions of EVs. Thus, SPECT imaging with ^99m^Tc-Duramycin-EVs may provide an approach with excellent sensitivity, easier quantification, and less impact on EV biological characteristics in EVs research and clinical translation.

In conclusion, our results showing differential involvement of liver cell types in EV uptake and release, may contribute to the development of improved EV-based drug delivery systems. ^99m^Tc-Duramycin-EVs holds promise as a unique method to track and measure EV homing and biodistribution systematically and noninvasively.

### Supplementary Information

Below is the link to the electronic supplementary material.

Supplementary file1 (PDF 946 KB)

## Data Availability

All data generated or analyzed during this study are included in this published article and its supplementary information file. We have submitted all relevant data of our experiments to the EV-TRACK knowledgebase (EV-TRACK ID: EV210178).

## Abbreviations

BSA: Bovine serum albumin
EV: Extracellular vesicle
FBS: Fetal bovine serum
FFA: Free fatty acid
GFP: Green fluorescent protein
HDL-C: High-density lipoprotein cholesterol
HEP: Hepatocyte
HFD: High fat diet
KC: Kupffer cell
LDL-C: Low-density lipoprotein cholesterol
lEV: Large-sized extracellular vesicle
LSEC: Liver sinusoidal endothelial cell
mEV: Medium-sized extracellular vesicle
NPC: Non-parenchymal cell
NTA: Nanoparticle tracking analysis
OA: Oleic acid
PA: Palmitic acid
PBS: Phosphate buffer saline
PFA: Paraformaldehyde
POt: Palmitic acid/oleic acid treatment
RT-qPCR: Real-time quantitative polymerase chain reaction
SD: Standard deviation
SEC: Size exclusion chromatography
sEV: Small-sized extracellular vesicle
TEM: Transmission electron microscope
TFF: Tangential flow filtration
VLDL-C: Very low-density lipoprotein cholesterol
