# Supplementary material for: Extracellular vesicle release and uptake by the liver under normo- and hyperlipidemia
Source: Cell Mol Life Sci. 2021 Oct 19;78(23):7589–604. doi: 10.1007/s00018-021-03969-6 (PMC8629784; doi:10.1007/s00018-021-03969-6)

Extracellular vesicle release and uptake by the liver under normo- and hyperlipidemia

Cellular and molecular life sciences

Krisztina Németh, Zoltán Varga, Dorina Lenzinger, Tamás Visnovitz, Anna Koncz, Nikolett Hegedűs, Ágnes Kittel, Domokos Máthé, Krisztián Szigeti, Péter Lőrincz, Clodagh O'Neill, Róisín Dwyer, Zhonglin Liu, Edit I. Buzás*, Viola Tamási*,†

† Corresponding author: Viola Tamási; Semmelweis University, Department of Genetics, Cell- and Immunobiology, Budapest, Hungary; viola.tamasi@gmail.com

* These authors share last authorship

Supplementary information

Supplementary Table 1 Details of antibodies used in experiments

	Manufacturer	Dilution	Lot	Catalog	Clone
**annexinV-Pacific Blue**	Sony Biotechnology	1:100	177485	3804590	-
**annexinV-AF647**		1:100	163910	3804715	-
**rat m. anti-mouse CD63-APC**		1:200	162405	1319530	NVG-2
**hamster m. anti-mouse CD81-PE**		1:400	104734	1124530	EAT-2
**mouse m. anti-human CD9-APC**		1:100	147840	2160540	HI9a
**mouse m. anti-human CD81-PerCP/Cy5.5**		1:100	150906	2347540	5A6
**mouse m. anti-human ApoB**	SantaCruz	1:100	E1418	sc-393636	A-6
**goat p. anti-mouse IgG-eF570**	ThermoFisher	1:100	2107466	41-4010-82	-
**mouse m. anti-human CD63-PerCP**		1:100	75080037	MA1-10269	MEM-259
**rabbit p. anti-human serum albumin**		1:100	VA2908285	PA5-85166	-
**goat p. anti-rabbit IgG-AF700**		1:100	2129003	A21038	-
**mouse m. anti-CD146**		1:100	2093219	14146982	P1H12
**rat m. anti-F4/80-eF660**		1:100	2056931	50480182	BM8
**TO-PRO-3 viability dye**		1:3000	1878895	T3605	-
**rat m. anti-mouse CD146-PerCP-Vio700**	Miltenyi Biotec	1:50	5191021526	130103795	ME-9F1
**recombinant m. human anti-mouse F4/80-PE**		1:100	5191002241	130116499	REA126
**anti-mouse FcR Blocking Reagent**		1:10	5191016254	130092575	-

Supplementary Fig. 1 Effect of the number centrifugations on murine PFP isolation

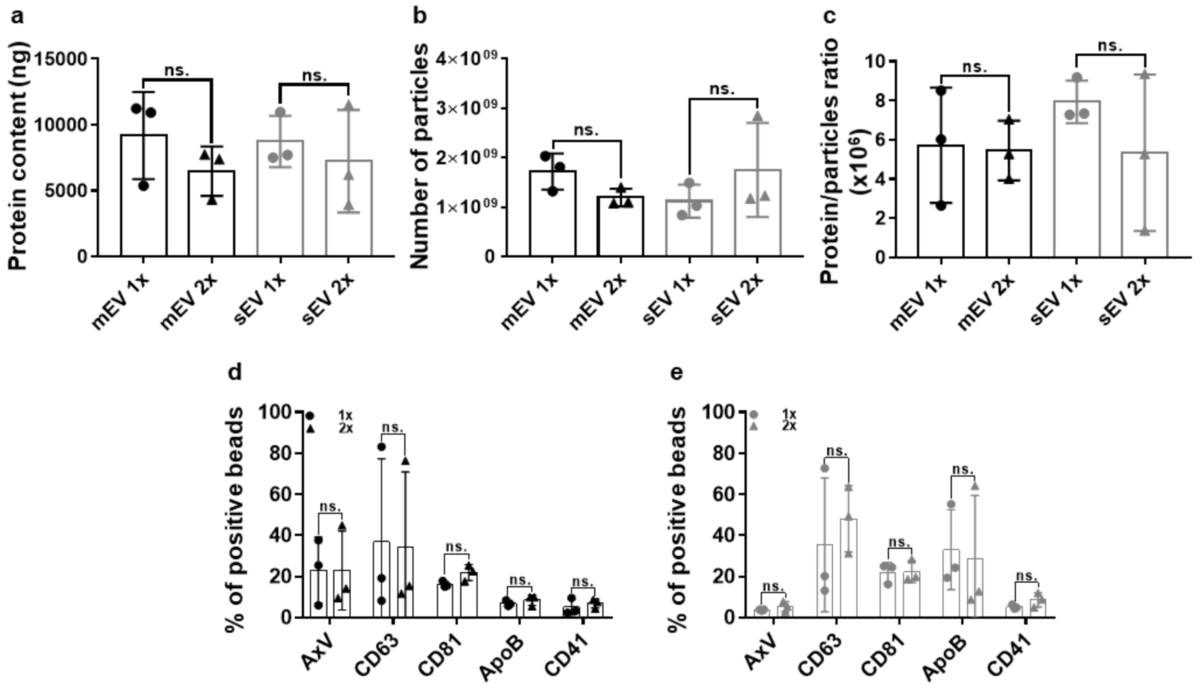

Mouse whole blood samples (500 µL) were centrifuged once or twice for plasma isolation. mEVs and sEVs were separated from plasma by a differential centrifugation combined with SEC. Based on protein and particle content (a and b), protein/particle ratio (c), EV (AxV, CD63, CD81, CD41) and non-EV (ApoB) markers (d and e), no difference was found between the EV content of plasma samples centrifuged once or twice. Values are reported as mean ± SD. p values were calculated by two-way ANOVA and Tukey's multiple comparisons post-hoc test. n= 3, ns: not-significant, mEVs (black symbols), sEVs (grey symbols), centrifuged once (circle symbols), centrifuge twice (triangle symbols)

Supplementary Table 2 Separation of plasma-derived EVs: detailed parameters of the centrifugation steps

	Centrifuge	Rotor	Tube	Centrifugation force (g)	Degree (°C)	Duration (min)	Accel	Decel
**Plasma**	HERMLE Z 216 MK	Angle Rotor 220.87	Eppendorf tubes 1.5 ml, PCR clean Ref. 0030125.215	1,500	4	15	Int.	Slow
**lEVs**				2,000		30		
**mEVs**				12,500		40		
**sEVs**	Beckman Coulter Optima MAX-XP Ultracentrifuge	MLA-55	ThermoFisher NuncCryoTubes (1.8 ml) Ref. 375418	100,000	4	70	Slow	Slow

Supplementary Fig. 2 The presence of plasma-derived mEVs (a) and sEVs (b) in the preparation was confirmed by TEM

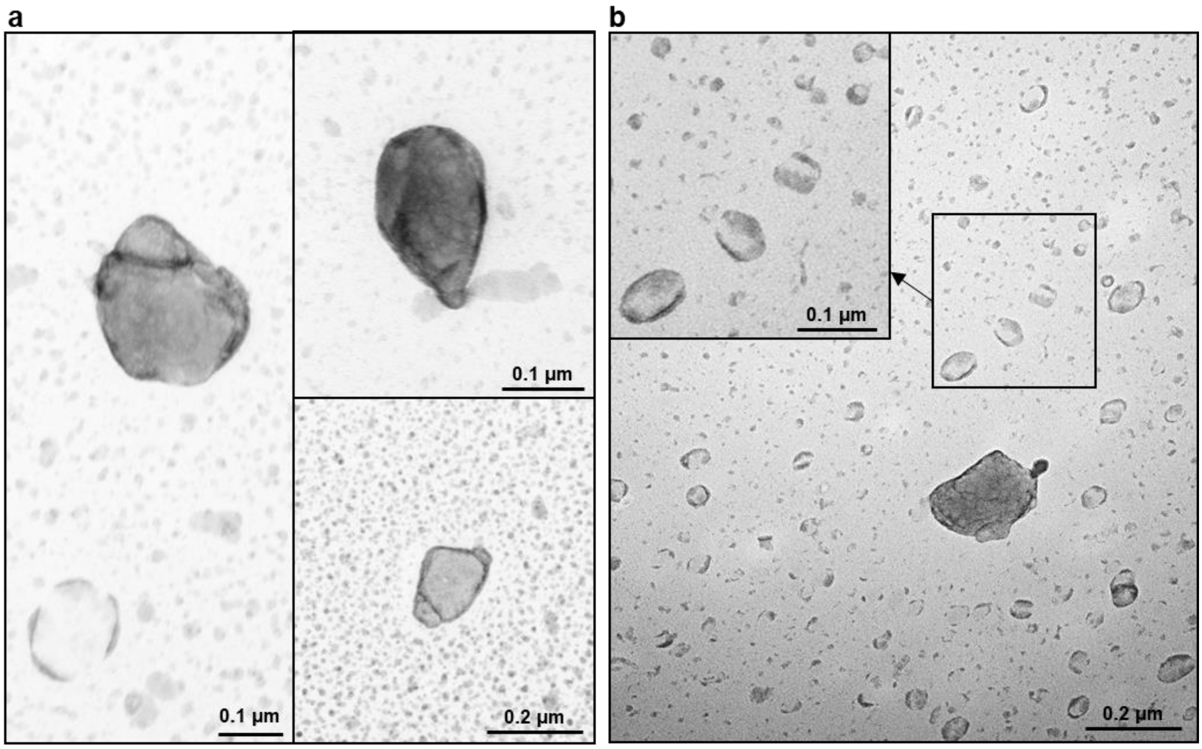

Supplementary Table 3 Separation of hepatocyte-derived EVs: detailed parameters of the centrifugation steps

	Centrifuge	Rotor	Tube	Centrifugation force (g)	Degree (°C)	Duration (min)	Accel	Decel
Cells	Eppendorf Centrifuge 5804 R	S-4-72	Sarstedt 15 ml conical Ref. 62.554.502	3,000	20	10	Int.	Int.
lEVs				2,000	4	30	Int.	Slow
mEVs	Eppendorf Centrifuge 5810 R	F-34-6-38		12,500	4	40	Int.	Slow
sEVs	Beckman Coulter Optima MAX-XP Ultracentrifuge	MLA-55	ThermoFisher NuncCryoTubes (1.8 ml) Ref. 375418	100,000	4	70	Slow	Slow

Supplementary Fig. 3 The presence of HEP-derived mEVs (a) and sEVs (b) in the preparation was confirmed by TEM

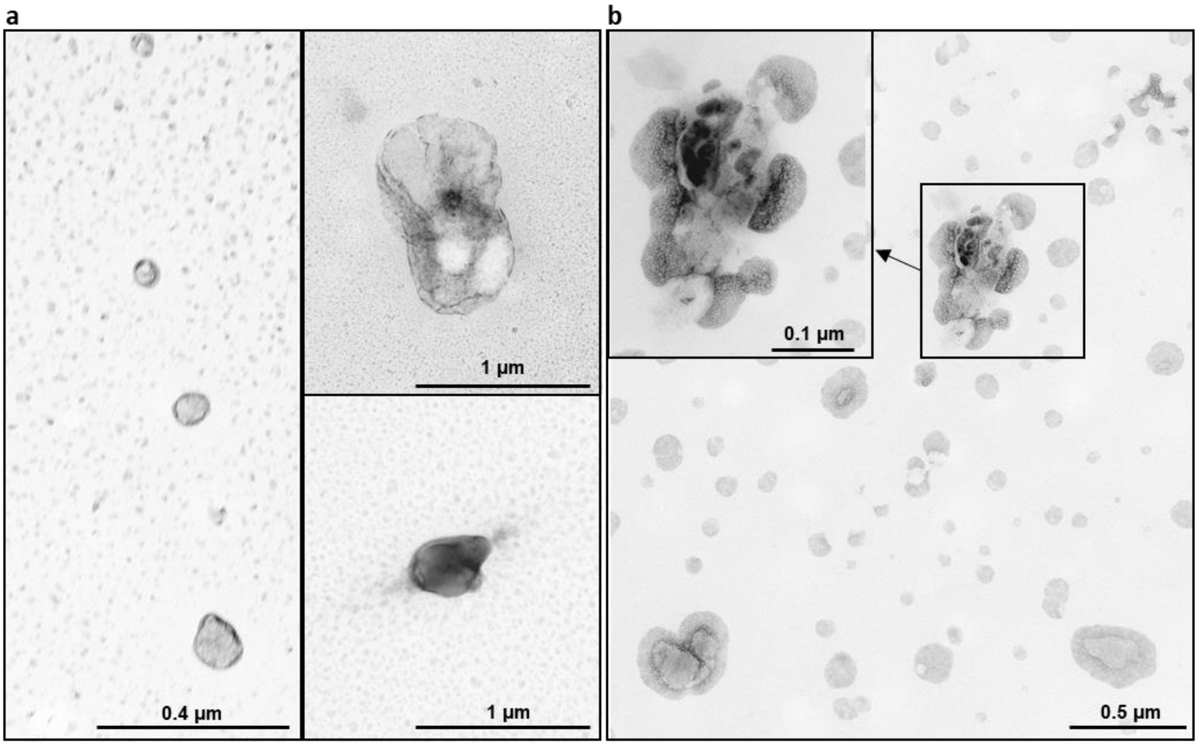

Supplementary Fig. 4 Assessment of the viability of HEK293T-palmGFP cells cultured for 24 h under serum free conditions

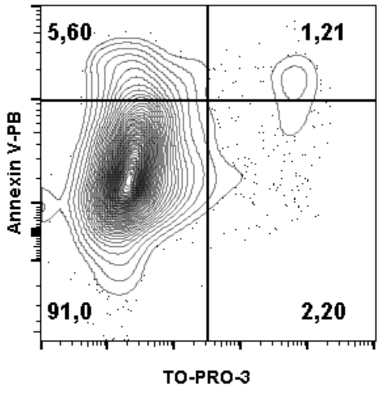

The viability of HEK293T-palmGFP cells were analysed by flow cytometry before EV separation. The proportion of apoptotic cells was determined by AxV and TO-PRO-3 stainings. Less than 7% of the cells are apoptotic.

Supplementary Fig. 5 The presence of HEK293T-palmGFP-derived mEVs in the preparation was confirmed by TEM

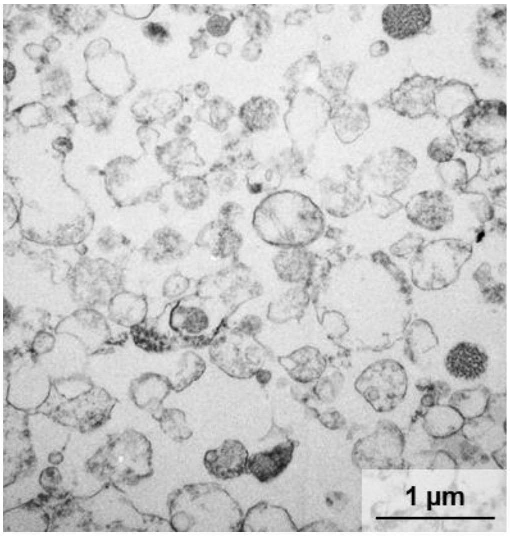

Supplementary Table 4 Separation of HEK293T-palmGFP-derived EVs: detailed parameters of the centrifugation steps

	Centrifuge	Rotor	Tube	Centrifugation force (g)	Degree (°C)	Duration (min)	Accel	Decel
**Cells**	Eppendorf Centrifuge 5804 R	S-4-72	Sarstedt 50 ml conical Ref. 62.559.001	300	20	10	Int.	Int.
**lEVs**				2,000	4	30	Int.	Slow
**mEVs**	Eppendorf Centrifuge 5810 R	F-34-6-38	Sarstedt 50 ml conical Ref. 62.547.254	12,500	4	40	Int.	Slow
**sEVs**	Beckman Coulter Optima MAX-XP Ultracentrifuge	MLA-55	ThermoFisher NuncCryoTubes (1.8 ml) Ref. 375418	100,000	4	70	Slow	Slow

Supplementary Table 5 Parameters of NTA measurements

	mEVs	sEVs
**Sensitivity**	60	85
**Shutter**	100	100
**FrameRate**	7,5	30
**Positions**	11	11
**Cycles**	2	2
**MinBrightness**	20	20
**MinSize**	5	5
**MaxSize (nm)**	1000	1000
**Temperature (°C)**	25	25

Supplementary Fig. 6 Identification of EV uptake by KCs and LSECs

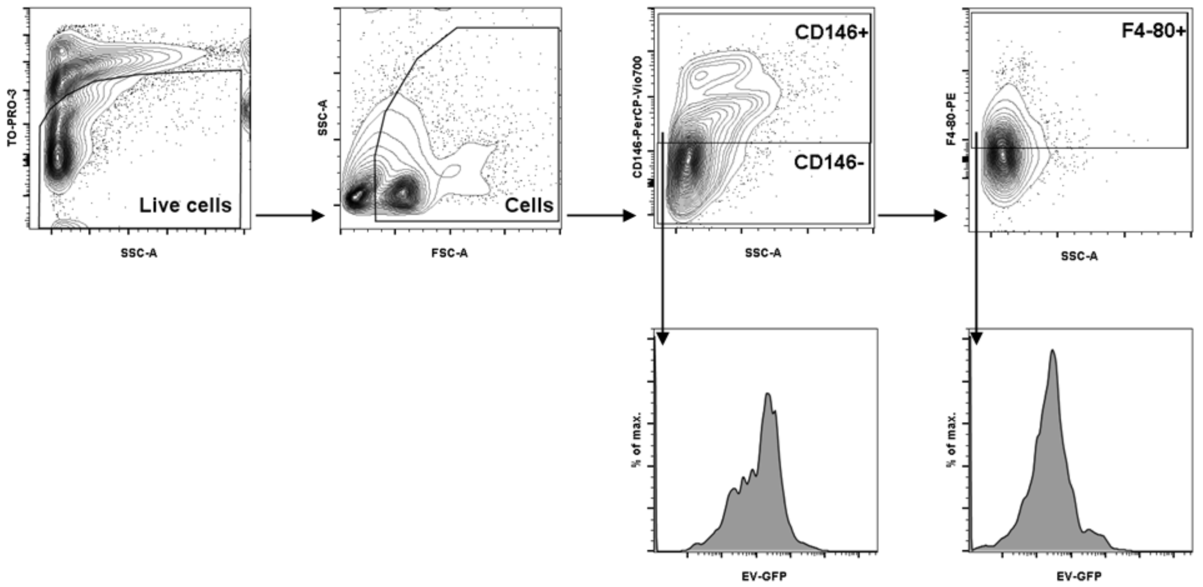

EV uptake of KCs and LSECs was examined by flow cytometry. TO-PRO-3 dye was used to identify live cells. The cells were separated from debris based on FSC, SSC values. CD146 was used to detect LSECs. Within CD146$^-$ cells KCs was identified as F4/80$^+$ cells.

Supplementary Fig. 7 Relative mean fluorescence intensity values of EV coated beads for plasma-derived mEVs (a)/sEVs (b and c) and hepatocyte-derived mEVs (d)/sEVs (e)

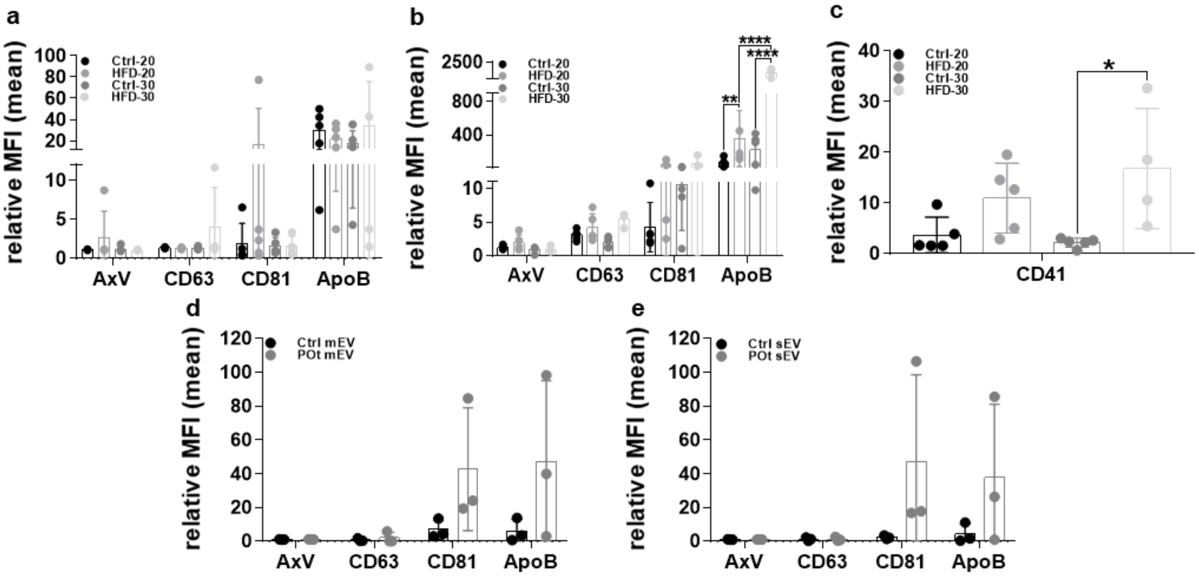

Supplementary Table 6 Proportion of positive beads in antibody control samples

	AxV %	ApoB %	CD63 %	CD81 %	CD41 %
**Plasma EVs**					
**mEV**	2,37	1,61	2,48	8,90	-
**sEV**	0,85	4,80	3,15	3,21	2,96
**Hepatocyte EVs**					
**mEV/sEV**	1,67	4,27	1,60	1,44	-

Supplementary Table 7 Median size (X50), particle concentration and protein/particle ratio of plasma-derived EVs

	Ctrl-20	HFD-20	Ctrl-30	HFD-30
Median (X50)				
mEV	223.8 ± 39.5	201.9 ± 29.1	246.6 ± 61.8	229.1 ± 33.3
sEV	108.0 ± 25.6	162.2 ± 48.2	137.1 ± 23.9	140.1 ± 16.2
Particles/mL				
mEV	9.9E7 ± 7.3E7	1.1E8 ± 3.6E7	2.4E8 ± 9.5E7	6.9E7 ± 3.1E7
sEV	5.0E9 ± 2.4E9	1.1E9 ± 2.6E8	2.1E9 ± 1.5E9	9.9E8 ± 2.5E8
Protein/particle				
mEV	14.6 ± 24.4	8.7 ± 3.7	4.8 ± 3.1	1.0 ± 1.3
sEV	17.7 ± 10.6	1.3 ± 0.5	8.4 ± 7.9	3.4 ± 3.8

Supplementary Table 8 Median size (X50) , particle concentration and protein/particle ratio of HEP-derived EVs

	Ctrl	POt
Median (X50)		
mEV	344.6 ± 26.5	319.9 ± 31.9
sEV	155.2 ± 4.6	150.9 ± 11.9
Particles/mL		
mEV	2.5E8 ± 8.4E7	1.5E9 ± 1.0E8
sEV	1.2E9 ± 2.3E8	6.4E9 ± 1.9E9
Protein/particle		
mEV	21.4 ± 8.9	9.0 ± 6.3
sEV	3.8 ± 1.0	2.4 ± 1.8

Supplementary Fig. 8 Average size of OilRedO+ particles (n=8)

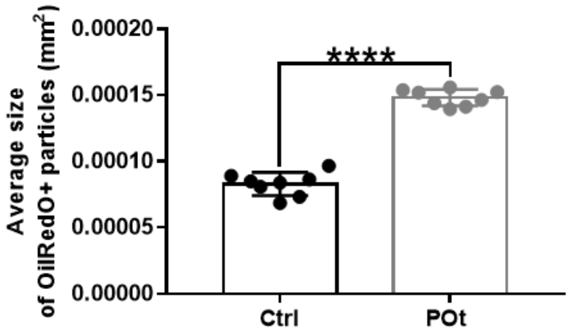

Supplementary Fig. 9 The ratio of KCs and LSECs in HEP-NPC co-culture (n = 3)

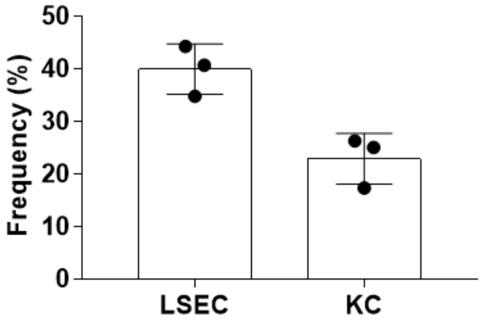

Supplementary Fig. 10 Effect of hyperlipidemia on liver cells cytokine production

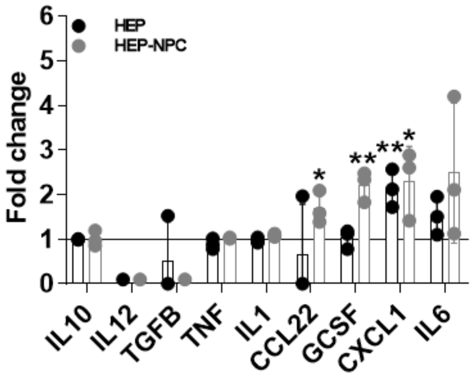

Cytokine production of the liver cells was analyzed from conditioned medium after 16h of FFA treatment. Values are reported as mean ± SD. p values were calculated by paired T-test. $n_{HEP} = 3$, $n_{HEP\text{-}NPC} = 3$, *<0.05, **<0.01

Supplementary Fig. 11 Proportion of positive beads in antibody control samples

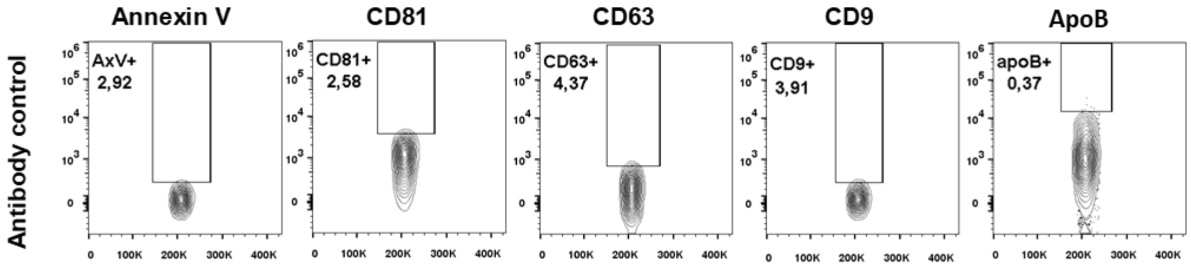

Antibody control samples were prepared as follows: the surface of the latex beads (without EVs) was blocked with 100 mM glycine and 2.5% w/v BSA solution. Afterwards, the beads were incubated with antibodies with the indicated dilutions (see in „Flow cytometry of EVs"). After washing out the excess antibodies, five thousand events/sample were measured by flow cytometry.

Supplementary Fig. 12 Lower power magnification micrographs of HEPs (a), KCs (b) and LSECs (c) in HEP-NPC co-culture

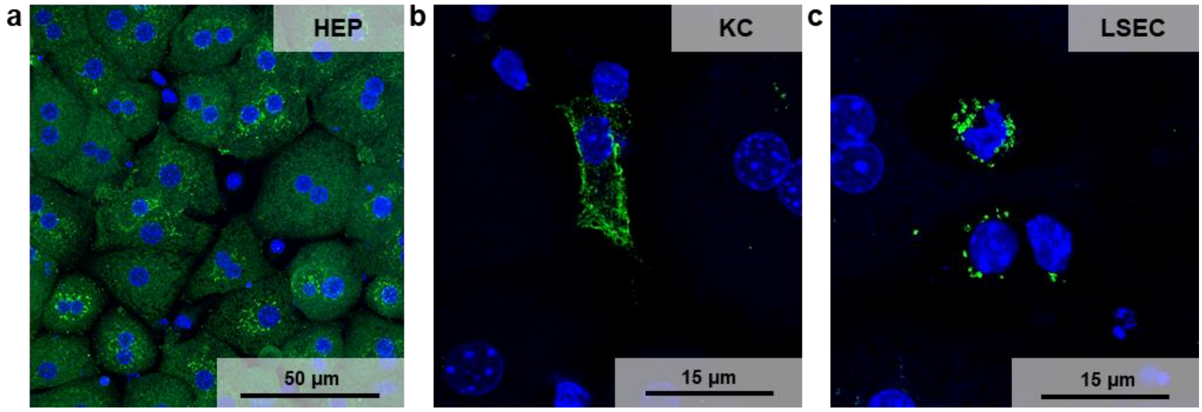

Supplement: Supplementary file 1 — Supplementary file1 (PDF 946 KB) [file 18_2021_3969_MOESM1_ESM.pdf]
